# Radiation-damage-induced phasing: a case study using UV irradiation with light-emitting diodes

**DOI:** 10.1107/S2059798315021658

**Published:** 2016-03-01

**Authors:** Daniele de Sanctis, Chloe Zubieta, Franck Felisaz, Hugo Caserotto, Max H. Nanao

**Affiliations:** a ESRF, The European Synchrotron, 71 Rue des Martyrs, 38000 Grenoble, France; b CNRS, Université Grenoble Alpes, CEA, DSV, INRA, iRTSV, Laboratoire de Physiologie Cellulaire and Végétale, UMR 5168, 38054 Grenoble, France; c European Molecular Biology Laboratory, 71 avenue des Martyrs, CS 90181, F-38042 Grenoble Cedex 9, France; d Unit of Virus Host Cell Interactions, UJF–EMBL–CNRS, UMI 3265, 71 avenue des Martyrs, CS 90181, F-38042 Grenoble Cedex 9, France

**Keywords:** light-emitting diodes, radiation damage, RIP, phasing, SIR, ultraviolet light, disulfide bonds, UV

## Abstract

Exposure to X-rays, high-intensity visible light or ultraviolet radiation results in alterations to protein structure such as the breakage of disulfide bonds, the loss of electron density at electron-rich centres and the movement of side chains. These specific changes can be exploited in order to obtain phase information. Here, a case study using insulin to illustrate each step of the radiation-damage-induced phasing (RIP) method is presented. Unlike a traditional X-ray-induced damage step, specific damage is introduced *via* ultraviolet light-emitting diodes (UV-LEDs). In contrast to UV lasers, UV-LEDs have the advantages of small size, low cost and relative ease of use.

## Abstract 2

A case study of radiation-damage-induced phasing is discussed using ultraviolet light-emitting diodes to induce specific radiation damage.

## 1. Introduction   

Radiation damage during an X-ray diffraction experiment occurs owing to the absorption of X-rays by electron-rich sites in the macromolecule, in certain cases resulting in damage to these specific sites preferentially (Burmeister, 2000 ▸; Ravelli & McSweeney, 2000 ▸; Leiros *et al.*, 2001 ▸). By tuning the dose of radiation, specific damage can be induced, creating the opportunity for *de novo* determination of the ‘substructure’ of radiation-damaged sites (Ravelli *et al.*, 2003 ▸) and phasing. This method of phasing was named radiation-damage-induced phasing (RIP) and can be exploited in a manner analogous to the single isomorphous replacement (SIR) method. RIP has now been used to determine phases in macromolecular crystals for over a decade (Evans *et al.*, 2003 ▸; Ravelli *et al.*, 2003 ▸, 2005 ▸; Banumathi *et al.*, 2004 ▸; Schiltz *et al.*, 2004 ▸; Weiss *et al.*, 2004 ▸; Zwart *et al.*, 2004 ▸; Ramagopal *et al.*, 2005 ▸). In RIP, the changes to structure factors either derive from the loss of electron density caused by radiation damage or the movement of existing atoms to new positions, for example changes in sulfur positions in disulfide-bond breakage. In contrast to the classical isomorphous replacement methods, which require a heavy-atom-derivatized and a native crystal, RIP can be performed using a single sample. One of the major limiting factors to the widespread use of RIP, however, is the degree of general radiation damage that is incurred during the course of the experiment. General radiation damage globally affects the crystal, often resulting in increased Wilson *B* factors, altered unit-cell parameters, increased mosaicity and decreased resolution (Burmeister, 2000 ▸; Ravelli & McSweeney, 2000 ▸). Since even small changes to unit-cell parameters have been known since the early days of isomorphous replacement to cause large changes in structure factors (Crick & Magdoff, 1956 ▸), this presents a serious problem for maximizing specific signal. Thus, for any radiation-damage-induced phasing experiment, one must maximize *specific* radiation damage and minimize *general* radiation damage by modulating the dose, which is often a challenging goal. To this end, various analytical and experimental techniques have been developed. The minimum acceptable general damage can be estimated from prior knowledge (Bourenkov & Popov, 2010 ▸) or determined empirically (Leal *et al.*, 2011 ▸), but assessing specific damage is more complex. Ancillary tools such as UV–visible or Raman spectroscopy are available at many synchrotron light sources, but can require specialized expertise in their operation (Carpentier *et al.*, 2010 ▸; Owen *et al.*, 2011 ▸, 2012 ▸). A balance between maximum specific damage and minimum general damage is also obtainable *post facto* (de Sanctis & Nanao, 2012 ▸). Even with these tools, however, this can be a difficult balance to achieve, and various methods have been developed in order to maximize the signal (owing to specific damage) to noise (owing to global damage) ratio. Furthermore, although specific damage that maximizes the RIP signal typically occurs around ∼2 MGy (de Sanctis & Nanao, 2012 ▸), it can be difficult to properly calculate X-ray absorbed dose, even with calibrated diodes (Owen *et al.*, 2009 ▸) and sophisticated new dose-calculation software (Zeldin *et al.*, 2013 ▸), particularly in the now common situation where the crystal is much larger than the beam. Often, the exposure time required to induce sufficient damage for phasing is overestimated, resulting in poor phases and difficult substructure solution. RIP using UV light has previously been shown to cause much less general radiation damage and to produce effects with a different mechanism from X-ray-induced damage. For these reasons, UV light offers an attractive alternative to X-ray-induced radiation damage. This technique has been termed ultraviolet radiation-damage-induced phasing (UV-RIP; Nanao & Ravelli, 2006 ▸). UV-RIP is typically performed with a laser at a wavelength of 266 nm. The primary source of differences in structure factors in UV-RIP is the disruption of disulfide and thioester bonds, as well as a reduction of the occupancy of some heavy atoms, such as selenium (de Sanctis *et al.*, 2011 ▸). While it has been shown that UV lasers are capable of inducing specific damage to macromolecular crystals, the technique can be limited by the costs associated with a dedicated UV laser setup, the technical requirements for precise laser-to-sample alignment, the attenuation of laser intensity owing to the use of fibre-optic cables and the specific safety requirements engendered by any laser experiment. Recently, however, high-power light-emitting diodes (UV-LEDs) at a variety of peak emissions in the UV range have become available at low cost. UV-RIP using UV lasers or diodes expands the repertoire of RIP techniques and provides an alternative to X-ray-induced radiation damage. Furthermore, UV-LEDs represent an excellent opportunity for the inexperienced RIP practitioner to experiment with the method because of their relatively low cost, high power and ease of alignment. Indeed, our results using these inexpensive and commercially available UV diodes gave similar results to previously published reports using more sophisticated UV laser setups. It should be noted that these results should also be achievable on home sources (Pereira *et al.*, 2013 ▸). Here, we use UV damage to insulin crystals by high-power UV-LEDs as a case study for RIP and provide a detailed workflow of a typical UV-RIP experiment.

## 2. Methods   

### 2.1. UV-LED support   

**Figure 1:**
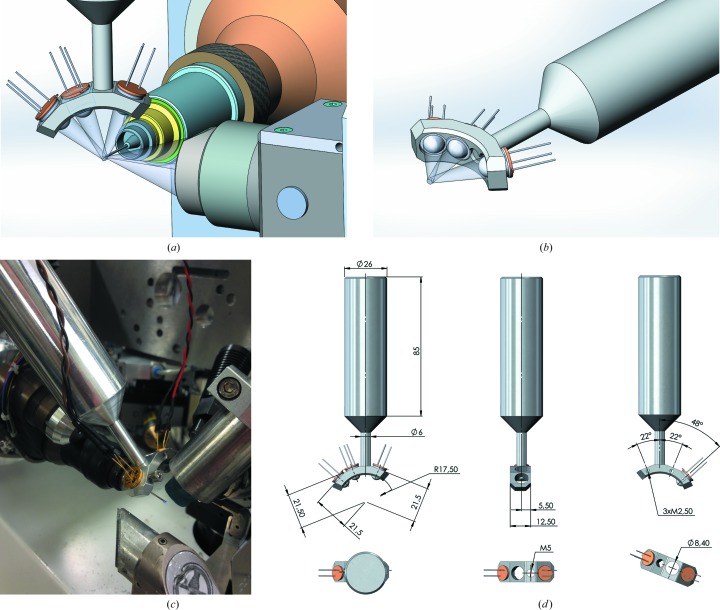
UV-LED support. (*a*, *b*) *CAD* drawings of the support, showing focal cones. (*c*) Installation on the ID29 beamline at ESRF. The LED light is focused to a spot by the built-in ball lenses. (*d*) Mechanical drawing of the LED support.

High-power UV-LEDs (Sensor Electronic Technology, USA) were obtained with built-in ball lenses of focal length 1.5–2.0 cm, a focal spot size of 1.5–2.0 mm and an emission maximum of 245 nm (UVTOP240TO39BL, Sensor Electronic Technology). UV illumination times were determined empirically. The 245 nm LED was initially chosen in the hope that it would yield different patterns of radiation damage compared with 266 nm UV lasers, but this was not found to be the case. We have designed a simple three-LED support with a shaft that fits into standard Oxford cryostream supports (Fig. 1 ▸); however, different configurations are possible depending on user specifications, sample environment and source design requirements. This design has a low enough volume to be compatible with the limited available space in the diffractometer environment. The support was positioned in order to allow the detector to be moved as close as possible to the diffractometer, and also to minimize shadow on the detector. Another approach to LED supports has previously been reported by Brayshaw *et al.* (2010 ▸), which uses a ring of LEDs that fits around a cryo-nozzle. While both solutions can adequately illuminate crystals, we believe that the independent control over the positioning of the spot afforded by a separate support and the larger unshaded rotation ranges of our design provide more flexibility for UV-RIP experiments. Independent movement does, however, have a downside in that installation is sure to be more time-consuming than with the Brayshaw design. Finally, the size of the Brayshaw design appears to be incompatible with the very tight space immediately surrounding the sample on the ID29 diffractometer (MD2).

LEDs were driven with a 24 V DC power supply, and two LEDs were connected in series with a 340 Ω resistor to give a forward current of 11.8 mA (half the maximum rated value). The power measured for a single 240 nm LED with a Fieldmaster GS (Cal Lab Technologies) power meter was 140 mW in a ∼1.5 mm spot for one LED. Two LEDS were used for these experiments, but the power meter was not easily adapted to measure the power for two LEDs simultaneously. While the absolute power is larger than previously measured power outputs from UV lasers (100 mW in a 150 µm spot; Nanao & Ravelli, 2006 ▸), the power density is a factor of ∼16 less. Proper alignment of the UV-LEDs with respect to the sample is required prior to data collection. This can be performed *via* observation of loop and paper fluorescence by direct visual observation *via* the on-axis camera of the diffractometer. During the alignment process, care should be taken to position the LEDs out of the trajectories of any diffractometer components and preferably in an orientation that casts a minimal or no shadow on the detector. Future experiments will use a pulsed source, which will allow eightfold higher currents. Our goal in these experiments was to determine whether phasing would be possible with LEDs, but systematic studies of the differences between UV-LED and UV laser phasing are envisioned.

### 2.2. Data-collection strategy   

In a UV-RIP experiment, several considerations must be considered. The first is to determine the suitability of the crystal for the technique. Although work is under way to identify other UV-sensitive groups, at present UV-RIP is limited to disulfide-, thioester- and selenium-containing proteins. The case is similar for X-ray RIP, but with the addition of a larger repertoire of heavy atoms and bound cofactors. Additionally, carboxylate damage by X-ray-induced radiation damage has on one occasion (R. G. B. Ravelli, unpublished work) produced a large enough signal for phasing but not for substructure solution. Thus, X-ray-induced damage appears to affect more sites, with the only requirement being relatively electron-rich sites, whereas UV damage may be more limited. However, this has not been systematically investigated. In both UV-RIP and X-ray RIP, crystals must be selected which are large enough to obtain two complete data sets. One issue specific to UV-RIP is that care must be taken to avoid selecting extremely large crystals. This is because, unlike X-rays, the UV penetration depth is typically of the order of a few micrometres (Nanao & Ravelli, 2006 ▸; Vernede *et al.*, 2006 ▸; Panjikar *et al.*, 2011 ▸). Although the specific values vary greatly with the contents of the unit cell, the amount of cryoprotection solution surrounding the crystal, the rate of photo-bleaching of UV-absorbing groups, the wavelength of the light used, the shape of the crystal and other parameters, smaller crystals will have a much larger percentage of their volumes irradiated than larger crystals. For example, cubic crystals with edges of 100, 50 or 10 µm, when uniformly irradiated from all sides, and a UV penetration depth of 5 µm will have roughly 27, 48 and 100% of their volumes irradiated (ignoring bleaching). It is therefore advisable to select the minimal crystal size possible in order to maximize the percentage of the crystal exposed to UV and reduce the amount of cryoprotectant solution. As in any RIP experiment, all data are collected from a single crystal to minimize non-isomorphism, since global radiation damage is an already significant source of non-isomorphism. Theoretical work has suggested that multi-crystal RIP is possible, at least in free-electron serial crystallography experiments with a very large number of crystals and a different damage mechanism (Galli *et al.*, 2015 ▸). However, to date such an approach has not been demonstrated to be possible using a small number of different crystals and synchrotron sources.

### 2.3. Insulin data collection   

In a normal UV-RIP experiment, a complete data set is collected, followed by UV exposure and the collection of a second complete data set. For our experiment, in order to show that it was UV damage rather than X-ray damage that was inducing specific changes, we collected an additional data set with the same parameters as the ‘after UV’ data set but without UV exposure. When comparing this data set with the first data set, minimal isomorphous differences should be observed if the X-ray dose is low enough. Each data set was collected at a new position on the crystal. Data-collection parameters were chosen that would yield complete data sets with a minimal X-ray radiation dose. A 45 × 45 × 45 µm cubic insulin crystal with space group *I*2_1_3 and unit-cell parameters *a* = *b* = *c* = 78.47 Å was cooled directly in the nitrogen stream at 100 K on ESRF beamline ID29 (de Sanctis *et al.*, 2012 ▸). Data were collected using a 10 µm cleaning aperture on a MD2 diffractometer (Maatel, Voreppe, France). Images were collected on a Pilatus 6M detector. The crystal was oriented diagonally relative to the goniometer axis, thus making ∼60 µm of crystal available. The ‘before’ data set was collected, followed by translation of the crystal by ∼15 µm and the collection of a second complete data set (Table 1 ▸). Note that this caused different crystal volumes to be irradiated, which resulted in small differences in dose between the different data sets (Table 1 ▸). The crystal was then moved by another 15 µm and the UV LEDs were then switched on for 10 min with the sample rotating to spread the UV damage over the crystal volume. A last data set was collected after UV exposure at this crystal position. The data-collection parameters were the same for all data sets. X-ray doses were calculated using *RADDOSE*-3*D* (Zeldin *et al.*, 2013 ▸).

## 3. Results   

The workflow for solving the radiation-damage substructure from UV-induced damage is analogous to the solution of any RIP substructure. A UV-RIP experiment can be broken down into four distinct phases: (i) experimental setup, (ii) maximization of the difference signal before and after UV exposure, (iii) substructure solution and (iv) phasing, as discussed below.

### 3.1. Difference signal   

A theoretical estimation based on the Crick–Magdoff equation (Crick & Magdoff, 1956 ▸) of the maximum possible signal owing to the breakage of disulfide or thioester bonds *via* UV-induced damage provides an idea of the possible signal which can be obtained for the experiment. The change in the magnitudes of the intensities before and after X-ray exposure has previously been estimated to be rather large at 10% for acentric reflections at 2θ = 0, given even modest (26%) reductions in sulfur occupancies (Ravelli *et al.*, 2003 ▸). However, it should be noted that these estimates provide the theoretical maximum signal and do not take into account non-isomorphism introduced by global radiation damage. In our insulin data sets, occupancies for S^γ^ positions were refined in *BUSTER* (Bricogne *et al.*, 2011 ▸) for the before and after data sets and were found to be reduced by up to 15% for Cys7 in both chain *A* and chain *B*, leading to expected differences of 19% at 2θ = 0. This position exhibited the highest difference in occupancy, with Cys19 exhibiting the smallest difference in occupancy. However, even knowing the protein structure, it is still not possible to predict the relative sensitivities of even well studied radiation-sensitive groups such as disulfide-bond sulfurs. Such estimates of predicted occupancy changes and predicted difference signal are also not available during *de novo* phasing. Instead, we rely on the average differences between the before and after data sets divided by their sigmas [〈*d*′/sig(*d*′)〉] binned by resolution to ascertain whether there will be sufficient signal for RIP. As with other phasing methods, these values should exhibit a downward trend with resolution, and the resolution for substructure determination is typically truncated to the resolution bin in which 〈*d*′/sig(*d*′)〉 falls below 1.3. The *R* value calculated between the before and after data sets can also be useful at this stage, with large values typically indicating an excess of non-isomorphism owing to global radiation damage. Both of these metrics, average differences and *R* values between data sets, can be useful for the pre-selection of data sets, particularly in the common case of multiple RIP experiments performed at, for example, different UV illumination times or X-ray burn times. However, the speed and user-friendliness of modern structure-determination programs often makes it advisable to continue with substructure determination, even if the previous metrics do not indicate a large amount of signal. For the UV-exposed data set (position 3) compared with position 1 (non-UV exposed), we observed differences of up to 1.83 in 〈*d*′/sig(*d*′) and these differences extended to 2.07 Å resolution. The *R*_isomorphous_ at low resolution (50.0–8.1 Å) was 10%. Although a model is not normally available for such a calculation, we used *ANODE* (Thorn & Sheldrick, 2011 ▸) to determine the model-phased *F*_before_ − *F*_after_ map peak heights, which were up to 22σ for both the position 3–position 1 and position 3–position 2 data sets. By contrast, 〈*d*′/sig(*d*′)〉 for position 2–position 1 reaches only 1.22 and only in the lowest resolution shell, and the model-phased *F*_before_ − *F*_after_ map peak heights reached a maximum of only 6.74σ (at the S^γ^ position of Cys7 in chain *B*) for this comparison. This suggested that there was significant UV damage between the UV-illuminated position and the two other data sets, while there was relatively little damage between the two ‘X-ray only’ data sets.

### 3.2. Substructure solution   

**Figure 2:**
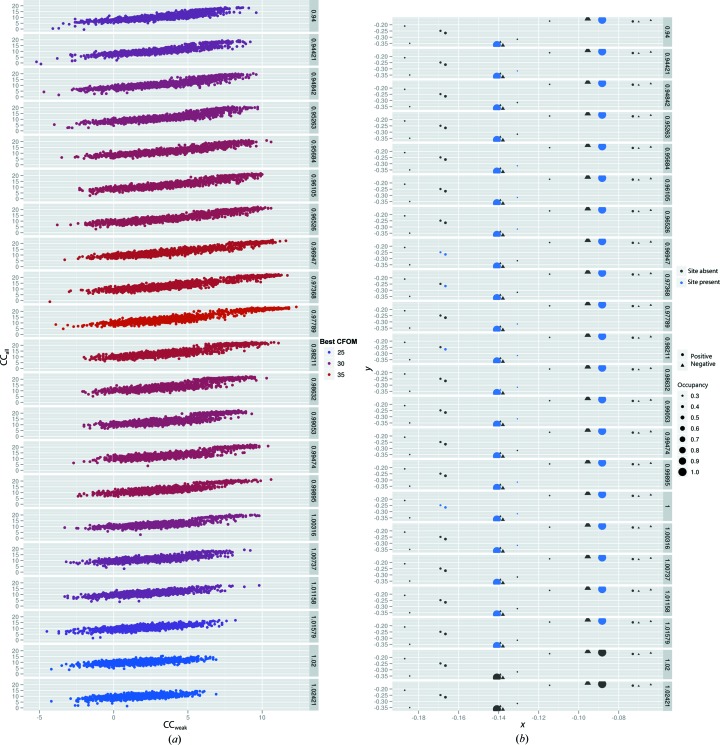
Success of *SHELXD* substructure solution. (*a*) *SHELXD* correlation coefficient for all reflections (CC_all_) *versus* weak reflections (CC_weak_) as a function of *k*. Sets of trial solutions are shown for multiple values of *k* and are coloured by the best combined figure of merit (CFOM) from that set of trials from blue to red. Note the maximum at *k* = 0.97789. (*b*) *Post facto* comparison of the best substructures from (*a*) against known substructures. Known substructures are computed by peak-searching a model-phased RIP difference map. The position and relative intensity of the resultant substructure sites are plotted in the *xy* plane of the unit cell. More intense sites (*i.e.* sites with the most radiation damage such as the S^γ^ atoms of some cysteines) are shown as larger circles. This reference substructure is then used to evaluate trial solutions from *SHELXD*. If a particular site is present in the *SHELXD* solution the circle is coloured blue, otherwise it is coloured grey. Note that the proportion of found (blue) sites is maximal at at *k* = 0.97789, like the best CFOMs.

It has previously been shown that perturbing the scale factor, *k*, of conventionally scaled before and after data sets by a small percentage can greatly improve the success rate of X-ray RIP in particular (Nanao *et al.*, 2005 ▸). Although the reasons for this are still not well understood, it appears to be related to the overall reduction in structure-factor intensities from global radiation damage. This is consistent with the fact that *k* scaling is less important for success in UV-RIP. The optimal *k* is determined empirically by varying it from 0.9 to 1.0 and comparing the statistics of the substructure-determination runs. Unfortunately, there is no single *k* value that works for all crystals, although the maximum for cases with strong signal is typically 0.97–0.99. Many powerful substructure-determination programs are now available, and almost any program that can solve SIR structures can be used for RIP. *SHELXD* is typically used for RIP both because of the ease with which it can be included in scripts and, more importantly, because *SHELXC*, its upstream partner, has been modified to include a keyword (DSCA) to modify *k* (Sheldrick, 2010 ▸). In all cases, the damaged data set is the ‘native’ and the undamaged data set is the ‘derivative’. As in any substructure determination by *SHELXD*, the presence of a well separated cluster of high CC_all_/CC_weak_ solutions is generally quite predictive of correct solutions, but these clusters tend to be less pronounced in a RIP experiment than in other methods (Fig. 2 ▸ *a*). For RIP, there is another metric that is highly predictive of success, which is the variation of the best combined figure of merit (CFOM) value in *SHELXD* *versus* *k*. The presence of a peak usually indicates that there is sufficient signal for phasing. One can see in Fig. 2 ▸ that the average and best solutions vary significantly, with a maximum at *k* = 0.97789. The position 2–position 1 data sets showed no such dependence of CFOM on *k*, as expected from the lack of signal. When one compares the substructures with the highest CFOM from each value of *k* with the known substructure (determined by peak-searching a model-phased *F*_beforeUV_ − F_afterUV_ map), one finds that the CFOM itself is also generally a reasonable indicator of substructure correctness (Fig. 2 ▸ *b*). However, it is worth noting that although the correlation coefficient-based metrics such as CC_all_/CC_weak_ and CFOM are still high above a *k* of 1, they are rarely correct (Fig. 2 ▸ *b*). As mentioned earlier, UV-RIP generally induces less overall radiation damage and thus it is sometimes possible to successfully determine partially correct substructures even without varying *k*. Despite this, it is still recommended to optimize this variable in a UV-RIP experiment if for nothing else other than to observe whether the CFOMs vary with *k*.

### 3.3. Phasing   

**Figure 3:**
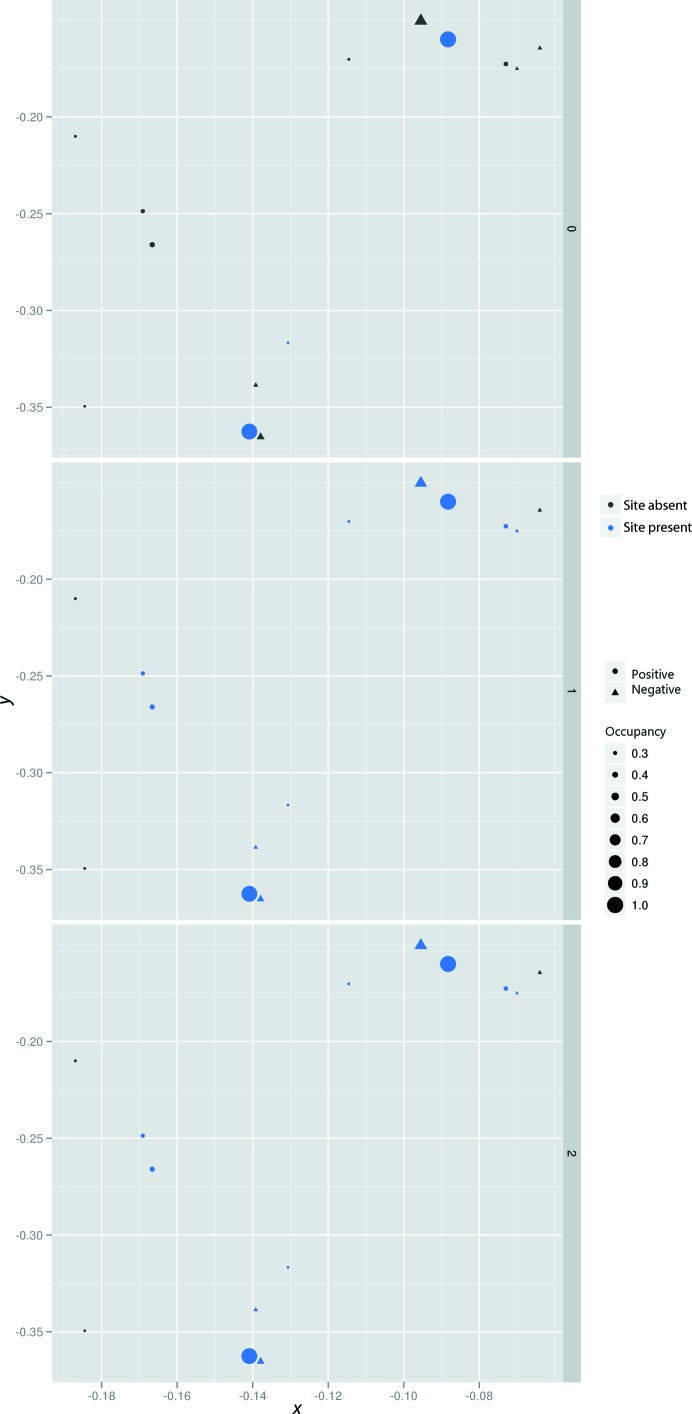
Analysis of difference Fourier maps allows the iterative improvement of RIP substructures. As in Fig. 2 ▸, a reference substructure is first computed. However, in this case the presence of negative sites (which come from atoms moving to new positions) is indicated by triangles. At each round of substructure improvement by difference Fourier analysis (indicated in the dark grey bar on the right), minor positive sites, which come from weakly damaged sites (circles) as well as negative sites (triangles), are identified (purple). The size of the shapes are scaled to their relative occupancies.

**Figure 4:**
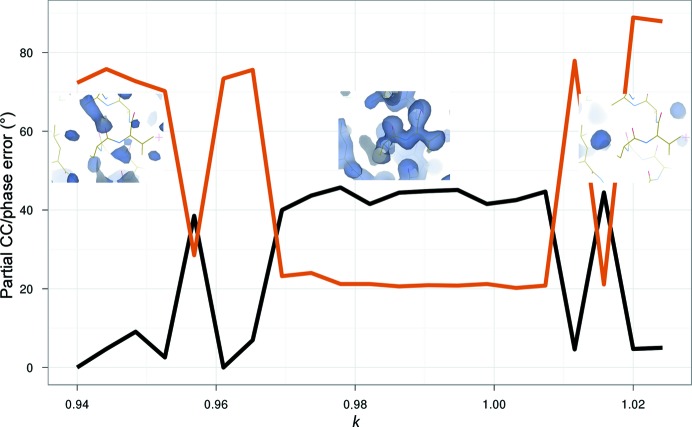
Correlation coefficient (CC) of the partially automatically built structure by *SHELXE* with native data (black) and figure-of-merit-weighted mean phase error (orange) *versus* *k*. Insets: electron-density maps superimposed on the final refined model are shown at several values of *k*.

Experimentally determined initial RIP substructures, even in the best of cases, are incomplete. This is because RIP substructures are comprised of a large number of relatively low-occupancy sites, as well as negatively occupied sites (owing to the movement of atoms to new positions). One solution exists to address both of these problems, which is to search isomorphous difference Fourier maps calculated from the initial partial substructures in order to identify lower occupancy and negative sites. This bootstrapping method is not unique to RIP, and is indeed used in both isomorphous and anomalous methods, but it is much more critical in RIP because of the prevalence of weak and negative sites. However, because initial RIP substructures are in general less complete than in other methods, and the success of bootstrapping depends heavily on the quality of the initial substructure and phase set, this can make the substructure-solution step more prone to failure. The iterative improvement of the insulin RIP substructure can be clearly seen in Fig. 3 ▸, where the correctness of the insulin substructure improves with successive rounds of peak-searching combined with phase improvement in *SHELXE*. In more demanding cases, for example in cases with weak signal and/or low resolution, it is frequently necessary to use *SHARP* at this stage (Schiltz & Bricogne, 2007 ▸). Once the substructure has been elaborated, as with single isomorphous replacement (McCoy & Read, 2010 ▸), both hands must be evaluated, since it is not possible to distinguish between the two based on phasing statistics. Generally, both hands are tried in parallel, for example by running *SHELXE* with and without the ‘-i’ flag. By adding information on the anomalous scattering owing to either the sulfurs or other electron-rich scatters which may be present in the sample (selenium for selenomethionine-derivatized proteins or the metal centres of metal-binding proteins, for example), the phase ambiguity can be broken by the additional anomalous scattering phasing information. In the more common case of RIP without anomalous signal, statistics derived from the electron density such as the contrast and CCs are identical between the correct and incorrect hands. However, the inclusion of automatic chain tracing with phase improvement in recent versions of *SHELXE* provides a powerful tool to not only determine which hand is correct but indeed to determine whether the structure is solved without the need to manually inspect an electron-density map. The key statistics that make this possible are the correlation coefficient between the partially automatically built model against the native data and the average chain length. If the former is >25% and the latter is >∼10 residues per fragment, the structure is often solved in that hand (Fig. 4 ▸). These empirically determined thresholds become less reliable at lower resolutions, however. In our insulin data, we observed partial CCs of up to 45%, with almost the entire molecule (51 residues) built, albeit as a single chain instead of two disulfide-linked chains. The final experimental electron-density map was readily interpretable and had a FOM-weighted phase error of 16.5° compared with a refined model.

## 4. Discussion   

Here, we demonstrate the steps necessary for RIP and show that UV-LEDs can be used to introduce sufficient specific radiation damage to determine phases. UV-LEDs are an inexpensive, easy-to-align and high-power alternative to UV lasers. They are versatile and simple enough to be incorporated into home-source systems and offer a method to induce radiation damage to the sample in a controlled and time-efficient manner compatible with virtually any diffraction setup. Indeed, the relative ease of inducing large differences in sulfur occupancies should be taken into consideration when using any technique that exposes crystals to UV light. Furthermore, UV-LEDs are available in a wide range of peak wavelengths, offering the exciting possibility of identifying different radiation-sensitive groups. The relatively wide spectral bandwidth results in a power output comparable to that of some UV lasers, and since our goal in using UV-LEDs is phasing, the increased power at the expense of bandpass is an acceptable compromise. UV-RIP and RIP in general proceed with the familiar steps used in other experimental phasing methods: analysis of the magnitude of the differences between reflections, substructure determination and finally phase calculation and improvement. While UV-RIP and RIP are closest in procedure to the single isomorphous replacement method, they differ in two key respects. Firstly, conventional scaling methods frequently overestimate the contribution of the damaged data set. This necessitates a slight downweighting of the damaged data set. Secondly, RIP substructures are generally comprised of many weak sites, and in many cases also contain negatively occupied sites. UV-RIP protocols reduce the number of weak sites somewhat; however, there are generally still many more weak sites than in an SIR experiment that has strong, but relatively few, sites in the substructure. These differences are not only of academic interest, but require the use of practical measures, specifically the rigorous iterative improvement of substructure by rounds of phase improvement and difference Fourier analysis. Despite these difficulties, RIP and UV-RIP also have some key advantages over SIR, including the potential for very high isomorphism, since the two data sets can be taken from the same crystal and indeed at the exact same position in the crystal, no requirement for direct chemical modification of the crystal and the ability to tune the amount of signal by changing the UV ‘burn’. Finally, UV-RIP can be combined with other methods such as long-wavelength sulfur SAD in the absence of heavy atoms (Rudiño-Piñera *et al.*, 2007 ▸) and indeed could be performed on the same crystal. We hope that the ease of use of UV-LEDs makes the adoption of UV-RIP more widespread in general.

## Figures and Tables

**Table 1:** Data-collection statistics Values in parentheses are for the outer shell.

	Before	Position 2	Position 3 (after UV)
Wavelength (Å)	0.9537	0.9537	0.9537
Resolution range	50–1.47 (1.52–1.47)	50–1.45 (1.50–1.45)	50–1.52 (1.57–1.52)
Space group	*I*2_1_3	*I*2_1_3	*I*2_1_3
Unit-cell parameters (Å)	*a* = *b* = *c* = 78.47	*a* = *b* = *c* = 78.47	*a* = *b* = *c* = 78.47
Total reflections	51661 (4682)	53754 (4963)	47100 (3746)
Unique reflections	13741 (1319)	14299 (1368)	12448 (1137)
Multiplicity	3.7 (3.5)	3.7 (3.6)	3.7 (3.3)
Completeness (%)	99.2 (99.2)	99.3 (99.2)	99.2 (99.1)
Mean *I*/σ(*I*)	11.58 (1.84)	10.03 (2.00)	11.01 (1.86)
Wilson *B* factor (Å^2^)	26.6	26.6	27.3
*R*_merge_	0.038 (0.573)	0.052 (0.541)	0.042 (0.5293)
CC_1/2_	0.997 (0.673)	0.994 (0.700)	0.998 (0.809)
Total exposure time (s)	14.0	14.0	14.0
Photon flux (photons s^−1^)	1.4 × 10^11^	1.4 × 10^11^	1.4 × 10^11^
Diffraction-weighted X-ray dose (MGy)	3.19	3.76	4.34
